# Machine learning–based prediction of suicide attempts among adolescents: a national study using explainable artificial intelligence

**DOI:** 10.3389/fpsyt.2026.1868329

**Published:** 2026-07-08

**Authors:** Eun Sun So, Ji-Young Yeo

**Affiliations:** 1 College of Nursing, Jeonbuk National University, Jeonju, Republic of Korea; 2 AI Institute, Hanyang University, Seoul, Republic of Korea

**Keywords:** adolescent, machine learning, prediction model, SHAP, suicide attempts

## Abstract

**Objectives:**

To develop and evaluate machine learning models for predicting adolescent suicide attempts and to examine predictor contributions using explainable artificial intelligence.

**Methods:**

A repeated cross-sectional study used pooled data from the 2017–2024 Korea Youth Risk Behavior Web-Based Survey (n=448, 798). Models including logistic regression, random forest, and XGBoost were developed to classify suicide attempts. Performance was evaluated using AUC, F1 score, and sensitivity-oriented screening thresholds to reflect population-level screening purposes. SHapley Additive exPlanations (SHAP) quantified predictor contributions.

**Results:**

The models showed moderate predictive performance, with XGBoost achieving the highest F1 score and random forest showing the highest sensitivity under the screening condition. SHAP analysis indicated that hopelessness contributed most strongly to prediction, followed by school violence and perceived stress. Additional contributors included self-rated health, household economic status, sleep satisfaction, and behavioral indicators. Under the screening condition, sensitivity improved, although positive predictive values remained low.

**Conclusion:**

Machine learning models demonstrated moderate performance in predicting adolescent suicide attempts. Psychological and social variables contributed most strongly to prediction, and SHAP improved the interpretability of model outputs, supporting their potential utility for population-level screening support.

## Introduction

Suicidal behaviors among adolescents represent a major public health concern worldwide and remain one of the leading causes of death in this population (1, 2). Suicide attempts are critical indicators of severe psychological vulnerability and are strongly associated with subsequent suicide death (3). Early identification of adolescents at high risk is therefore essential for effective prevention and intervention.

A wide range of factors across demographic and family, physical and health status, psychological, health behavioral, circadian and digital, and social and school-related domains have been associated with adolescent suicide attempts. First, demographic and family factors have shown consistent associations with suicide risk. Demographic characteristics such as sex and school grade have shown consistent associations, with female adolescents and lower school grades reporting higher rates of suicide attempts (4, 5). Temporal factors, including broader societal changes such as the COVID-19 pandemic, have also been linked to variations in adolescent suicide risk (5). Socioeconomic disadvantage and family structure, particularly not living with both parents, have been identified as important determinants of suicide risk (5–7). Regional differences, such as urban versus rural residence, have been examined in relation to contextual influences (5, 7).

Second, physical and health status factors have also been associated with suicidal behaviors. While objective indicators such as body mass index have shown inconsistent findings, subjective measures including perceived body image and self-rated health have demonstrated more consistent associations with suicide attempts (8–10). Third, psychological factors, particularly hopelessness and perceived stress, have been consistently identified as strong predictors of suicide attempts among adolescents (4, 5, 11, 12). Fourth, health behavioral, circadian, and digital factors further contribute to suicide risk. Early initiation of alcohol use and smoking has been associated with increased vulnerability to suicide attempts (11, 13, 14). Sleep-related factors, including sleep satisfaction, have also been linked to suicidal behaviors, although findings remain inconsistent (15, 16). Circadian characteristics, such as chronotype and social jetlag, have recently been examined in relation to emotional regulation and suicide attempts (16). In addition, digital behaviors, including daily internet use time, have been associated with suicidal behavior outcomes, although their relationship with suicidal behaviors remains complex (17). Finally, social and school-related factors have also been consistently associated with suicidal behaviors. Exposure to school violence has been identified as a significant risk factor for suicide attempts (4, 6), and academic performance has been examined as a potential correlate of adolescent suicide attempts.

Most previous studies have relied on conventional statistical approaches, such as logistic regression, to examine associations between these factors and suicidal behaviors. While these methods are useful for estimating independent effects, they are limited in capturing complex relationships among variables.

Recently, machine learning approaches have been increasingly applied in suicide research because of their ability to identify complex patterns across multidomain risk factors and improve the prediction of high-risk individuals (18, 19). In particular, machine learning models can capture nonlinear and interactive relationships among psychological, behavioral, social, physical health, circadian, and digital behavioral factors that may not be sufficiently reflected in conventional analytical approaches. These strengths may enhance the early identification of adolescents at elevated risk for suicide attempts.

In addition, explainable artificial intelligence techniques such as SHapley Additive exPlanations (SHAP) have recently enabled interpretation of machine learning models by quantifying the relative contribution and importance of individual predictors while maintaining predictive performance (20). This approach may improve the applicability of machine learning models in public health and suicide prevention research by simultaneously providing predictive accuracy and interpretability.

However, previous machine learning studies on adolescent suicide prediction have often focused on limited sets of predictors or specific high-risk populations. In contrast, the present study applied a comprehensive multidomain framework within a nationally representative adolescent population, incorporating demographic and family, physical health and health status, psychological, health behavioral, circadian and digital behavioral, and social and school-related factors. Furthermore, this study integrated machine learning–based prediction, screening-oriented threshold evaluation, and SHAP-based interpretability to provide a more comprehensive and explainable framework for adolescent suicide attempt prediction.

Therefore, this study aimed to evaluate predictive performance using machine learning models and assess the relative importance of predictors using SHAP to develop an explainable framework for adolescent suicide attempt prediction.

## Methods

### Design and population

This study was a repeated cross-sectional study using pooled data from the 2017–2024 Korea Youth Risk Behavior Web-Based Survey (KYRBS) (21). The KYRBS is an annual, anonymous, self-administered online survey conducted by the Korea Disease Control and Prevention Agency in collaboration with the Ministry of Health and Welfare and the Ministry of Education of Korea. The survey targets middle and high school students (grades 7–12) and is designed to assess health-risk behaviors among Korean adolescents. The KYRBS uses a stratified, multistage cluster sampling design to obtain a nationally representative sample, with stratification based on geographic region and school type, followed by random selection of schools and classes. For the present study, data from the 2017–2024 survey cycles were pooled, resulting in a final analytic sample of 448, 798 adolescents.

### Measurements

The primary outcome of this study was suicide attempt. Suicide attempt was assessed by asking whether participants had attempted suicide during the past 12 months (yes/no).

Independent variables were categorized into six domains: demographic and family, physical health and health status, psychological, health behavioral, circadian and digital behavioral, and social and school-related domains.

#### Demographic and family factors

included sex, grade, survey year, household economic status, city type, and family structure. Sex was categorized as male or female. Grade ranged from middle school 1st grade to high school 3rd grade. Survey year ranged from 2017 to 2024. Household economic status was assessed using a five-point self-reported scale (very high, high, moderate, low, very low). City type was classified into metropolitan city, small- and medium-sized city, and rural area based on the regional classification provided in the KYRBS dataset. Family structure was categorized into three groups: living with both parents, living with a single parent, and living without parents.

#### Physical and health status factors

included height (z-score), body mass index (BMI) (z-score), perceived body image, and self-rated health. Height and weight were self-reported and used to calculate BMI as weight in kilograms divided by height in meters squared. Both height and BMI were standardized into z-scores based on age- and sex-specific reference growth charts. Perceived body image was assessed using a five-category scale (very thin, thin, normal, overweight, very overweight). Self-rated health was measured using a five-point Likert scale ranging from very unhealthy to very healthy.

#### Psychological factors

included hopelessness and stress. Hopelessness was assessed by whether participants experienced sadness or hopelessness severe enough to interfere with daily activities for at least two weeks during the past 12 months (yes/no). Stress was measured using a five-point Likert scale ranging from very high to very low.

#### Health behavior factors

included age at first alcohol use, age at first cigarette smoking, and sleep satisfaction. Age at first alcohol use and cigarette smoking were originally assessed in school-grade categories and converted into age in years. Sleep satisfaction was measured using a five-point Likert scale indicating whether sleep during the past 7 days was sufficient for fatigue recovery.

#### Circadian and digital behavior factors

included chronotype, social jetlag, and daily internet use time. Chronotype was calculated as the midpoint of sleep on free days (MSF), derived from self-reported sleep onset and wake times on weekends, a commonly used indicator of chronotype in sleep and circadian rhythm research (16). Social jetlag was defined as the absolute difference between the midpoint of sleep on school days and free days, calculated from self-reported weekday and weekend sleep schedules (16). Daily internet use time was derived from self-reported sedentary time for non-academic purposes and measured in minutes per day.

#### Social and school-related factors

included experience of school violence and academic performance. Experience of school violence was assessed based on the number of incidents of violence or bullying requiring medical treatment during the past 12 months, and was measured as an ordinal variable with seven categories (0, 1, 2, 3, 4, 5, and ≥6 times). Academic performance was self-reported and categorized into five levels (very high, high, moderate, low, and very low).

### Data analysis

All statistical analyses incorporated complex sample weights provided by the KYRBS survey design to account for the stratified multistage sampling structure and to preserve national representativeness. Categorical variables were presented as weighted frequencies and percentages, and continuous variables were presented as weighted means and standard deviations. Group differences between adolescents with and without suicide attempts were assessed using Rao–Scott chi-square tests for categorical variables and complex sample general linear models for continuous variables.

Multivariable logistic regression analyses were conducted to examine the associations between independent variables and suicide attempts. Ordinal variables were treated as continuous variables in the regression analyses, and odds ratios were interpreted per one-level increase. Higher values of ordinal variables were coded to indicate greater severity or risk. Adjusted odds ratios (ORs) and 95% confidence intervals (CIs) were estimated.

For predictive modeling, the analytic dataset was randomly divided into training (80%) and test (20%) sets using stratified sampling based on suicide attempt status to preserve class distribution. A fixed random seed (random_state = 42) was applied to ensure reproducibility. Predictive performance was evaluated by comparing a conventional logistic regression model with machine learning models, including Random Forest and Extreme Gradient Boosting (XGBoost), using the area under the receiver operating characteristic curve (AUC). Because suicide attempts were relatively infrequent, model performance was additionally assessed using metrics sensitive to class imbalance, including recall and F1-score. Optimal classification thresholds were determined based on the F1 score. A sensitivity-oriented threshold (recall ≥ 0.70) was additionally applied to reflect the screening purpose of identifying high-risk adolescents, where minimizing false negatives was prioritized over overall classification accuracy. In suicide prevention contexts, failing to identify high-risk individuals may have greater consequences than increasing false positives (22).

To further assess the relative contribution of each variable, SHapley Additive exPlanations (SHAP) values were calculated, and feature importance was quantified as the mean absolute SHAP value (17). SHAP is an explainable artificial intelligence method derived from cooperative game theory that quantifies the contribution of each predictor to model predictions. Higher absolute SHAP values indicate a greater influence on the prediction outcome.

All analyses were conducted using SPSS (version 26; IBM Corp., Armonk, NY, USA) for complex sample descriptive analyses and Python (version 3.10) in Google Colaboratory for machine learning and predictive modeling. Python libraries included pandas (23), NumPy (24), scikit-learn (25), and XGBoost (26).

## Results

### Characteristics of participants according to suicide attempt

Most variables, except for city type, differed significantly between adolescents with and without suicide attempts based on Rao–Scott chi-square tests and complex sample general linear models (**Table 1**). Adolescents who reported suicide attempts were more likely than those without attempts to be female, to be in middle school (grades 7–9), to be surveyed in years other than the COVID-19 period (2020–2022), to have lower household economic status, and to live without both parents. In terms of physical and health status factors, adolescents with suicide attempts showed slightly higher height and BMI, more extreme perceived body image (i.e., perceiving themselves as either obese or very thin), and poorer self-rated health. Psychologically, they were much more likely to report hopelessness and higher levels of stress. Regarding health behaviors, they reported earlier initiation of alcohol use and smoking and lower sleep satisfaction. For circadian and digital behavior factors, they exhibited a later chronotype, greater social jetlag, and longer daily internet use time. In social and school-related factors, they were more likely to experience school violence and to have lower academic performance.

**Table 1:** Characteristics of adolescents according to suicide attempt.

Variable		No attempt (n=436, 570)	Attempt (n=12, 228)	t / χ²	p-value
Demographic and family factors					
Sex	Male	225490(52.2)	4448(37.8)	972.98	<.001
	Female	211080(47.8)	7780(62.2)		
Grade	7^th^	76303(16.3)	2265(17.4)	252.88	<.001
	8^th^	75853(16.3)	2495(19.3)		
	9^th^	75626(16.3)	2435(19.2)		
	10^th^	70910(16.8)	1667(14.1)		
	11^th^	70550(17.0)	1732(14.8)		
	12^th^	67328(17.3)	1634(15.2)		
Survey year	2017	60642(14.0)	1634(13.5)	242.90	<.001
	2018	58167(13.1)	1873(15.2)		
	2019	55572(12.4)	1731(13.7)		
	2020	53827(12.3)	1121(9.2)		
	2021	53603(12.2)	1245(10.0)		
	2022	50457(12.0)	1393(11.8)		
	2023	51186(11.9)	1694(14.1)		
	2024	53116(12.1)	1537(12.4)		
Household economic status	Very low	8920(2.0)	956(7.6)	2526.96	<.001
	Low	44097(9.8)	2074(16.2)		
	Moderate	206041(46.8)	4835(39.2)		
	High	128782(30.1)	2882(24.2)		
	Very high	48718(11.4)	1480(12.7)		
City type	Metropolitan city	190337(42.1)	5236(41.6)	3.64	.281
	Small- and medium-sized city	212963(52.1)	6095(52.9)		
	Rural area	33270(5.8)	897(5.5)		
Family structure	Both parents	348979(94.8)	9193(89.4)	1194.14	<.001
	Single parent	18144(4.6)	768(7.1)		
	Neither	2733(0.7)	351(3.5)		
Physical and health status factors					
Height (z-score)		0.35 ± 0.02	0.37 ± 0.03	-2.31	.021
BMI (z-score)		0.03 ± 0.12	0.07 ± 0.14	-2.83	.005
Body image	Very obese	30347(6.7)	1461(11.5)	621.66	<.001
	Obese	137770(31.5)	4118(33.7)		
	Normal	156381(35.9)	3740(30.6)		
	Thin	92519(21.4)	2191(18.2)		
	Very thin	19553(4.5)	718(6.0)		
Self-rated health	Very unhealthy	2009(0.5)	509(4.3)	6269.04	<.001
	Unhealthy	32234(7.5)	2346(19.1)		
	Normal	102245(23.6)	3909(31.6)		
	Healthy	191225(43.8)	3648(29.9)		
	Very healthy	108857(24.7)	1816(15.1)		
Psychological factors					
Hopelessness	Yes	110787(25.4)	9549(78.0)	16513.26	<.001
	No	325783(74.6)	2679(22.0)		
Stress	Very high	43023(9.9)	5097(41.5)	13940.82	<.001
	High	122016(28.0)	4257(34.6)		
	Moderate	188285(43.3)	2110(17.5)		
	Low	68302(15.4)	482(4.1)		
	Very low	14944(3.4)	282(2.3)		
Health behavior factors					
Age at first drinking		12.65 ± 0.12	11.45 ± 0.46	25.94	<.001
Age at first smoking		12.66 ± 0.13	10.56 ± 0.67	16.40	<.001
Sleep satisfaction	Very inadequate	59317(14.0)	3708(30.6)	2961.54	<.001
	Inadequate	125051(29.0)	3704(30.1)		
	Moderate	143245(32.6)	3075(25.2)		
	Adequate	75574(17.0)	1138(9.2)		
	Very adequate	33383(7.4)	603(4.9)		
Circadian and digital behavior factors					
Chronotype (MSF)		6.39 ± 0.01	6.90 ± 0.04	-13.60	<.001
Social jetlag (SJL)		2.16 ± 0.01	2.44 ± 0.03	-8.97	<.001
Daily internet use time		273.59 ± 0.72	354.38 ± 2.92	-28.29	<.001
Social and school-related factors					
Experience of school violence (times)		0.04 ± 0.00	0.52 ± 0.02	-23.51	<.001
Academic performance	Very low	41300(9.5)	2313(18.9)	1433.48	<.001
	Low	97599(22.3)	3159(25.4)		
	Moderate	129924(29.8)	3002(24.6)		
	High	110400(25.3)	2270(18.6)		
	Very high	57338(13.1)	1483(12.6)		

### Multivariable logistic regression analysis for suicide attempt

**Table 2** presents the results of the multivariable logistic regression analysis for suicide attempt. Female sex (OR = 1.45, 95% CI: 1.44–1.46, p < 0.001), lower grade (OR = 0.86, 95% CI: 0.86–0.86, p < 0.001), earlier survey year (OR = 1.00, 95% CI: 0.99–1.00, p < 0.001), and lower household economic status (OR = 1.07, 95% CI: 1.07–1.08, p < 0.001) were associated with higher odds of suicide attempts. Adolescents living in metropolitan (OR = 1.11, 95% CI: 1.09–1.12) and small- and medium-sized cities (OR = 1.07, 95% CI: 1.06–1.08) had higher odds compared to those in rural areas (all p < 0.001), and those not living with both parents also showed higher odds (OR = 1.03, 95% CI: 1.02–1.04, p < 0.001). Height (z-score) (OR = 1.00, 95% CI: 0.99–1.00, p = 0.006) was statistically significant but showed minimal association with suicide attempts. Compared to adolescents with normal body image, those who perceived themselves as very thin (OR = 1.25, 95% CI: 1.23–1.26), obese (OR = 1.08, 95% CI: 1.08–1.09), or very obese (OR = 1.22, 95% CI: 1.21–1.24) had higher odds of suicide attempts, whereas those perceiving themselves as thin showed slightly lower odds (OR = 0.98, 95% CI: 0.97–0.98) (all p < 0.001). Poorer self-rated health was associated with higher odds of suicide attempts (OR = 1.24, 95% CI: 1.24–1.25, p < 0.001). Hopelessness (OR = 5.37, 95% CI: 5.33–5.40, p < 0.001) and higher levels of stress (OR = 1.63, 95% CI: 1.62–1.64, p < 0.001) were associated with increased odds of suicide attempts. Earlier age at first alcohol use (OR = 1.07, 95% CI: 1.07–1.07, p < 0.001), earlier age at first smoking (OR = 1.10, 95% CI: 1.10–1.10, p < 0.001), and lower sleep satisfaction (OR = 1.03, 95% CI: 1.02–1.03, p < 0.001) were associated with higher odds of suicide attempts. A later chronotype (OR = 1.02, 95% CI: 1.02–1.02, p < 0.001) and longer daily internet use time (OR = 1.00, 95% CI: 1.00–1.00, p < 0.001) were associated with higher odds of suicide attempts, whereas greater social jetlag (OR = 0.99, 95% CI: 0.98–0.99, p < 0.001) was associated with slightly lower odds. A higher frequency of school violence experiences (OR = 1.62, 95% CI: 1.62–1.62, p < 0.001) and lower academic performance (OR = 1.06, 95% CI: 1.05–1.06, p < 0.001) were associated with increased odds of suicide attempts.

**Table 2:** Multivariable logistic regression analysis for suicide attempt.

Domain	Variable	OR	95% CI	p-value
Demographic factors	Sex (female vs male)	1.45	1.44–1.46	<0.001
	Grade	0.86	0.86–0.86	<0.001
	Survey year	1.00	0.99–1.00	<0.001
	Economic status	1.07	1.07–1.08	<0.001
	City type (medium/small vs rural)	1.07	1.06–1.08	<0.001
	City type (metropolitan vs rural)	1.11	1.09–1.12	<0.001
	Family structure	1.03	1.02–1.04	<0.001
Physical and health status factors	Height (z-score)	1.00	0.99–1.00	0.006
	BMI (z-score)	1.00	0.99–1.00	0.229
	Body image (Thin vs. Normal)	0.98	0.97–0.98	<0.001
	Body image (Very thin vs. Normal)	1.25	1.23–1.26	<0.001
	Body image (Obese vs. Normal)	1.08	1.08–1.09	<0.001
	Body image (Very obese vs. Normal)	1.22	1.21–1.24	<0.001
	Self-rated health	1.24	1.24–1.25	<0.001
Psychological factors	Hopelessness	5.37	5.33–5.40	<0.001
	Stress	1.63	1.62–1.64	<0.001
Health behavior factors	Age at first drinking	1.07	1.07–1.07	<0.001
	Age at first smoking	1.10	1.10–1.10	<0.001
	Sleep satisfaction	1.03	1.02–1.03	<0.001
Circadian and digital behavior factors	Chronotype (MSF)	1.02	1.02–1.02	<0.001
	Social jetlag	0.99	0.98–0.99	<0.001
	Daily internet use time	1.00	1.00–1.00	<0.001
Social and school-related factors	Experience of school violence	1.62	1.62–1.62	<0.001
	Academic performance	1.06	1.05–1.06	<0.001

### Predictive performance of machine learning models

**Figure 1:**
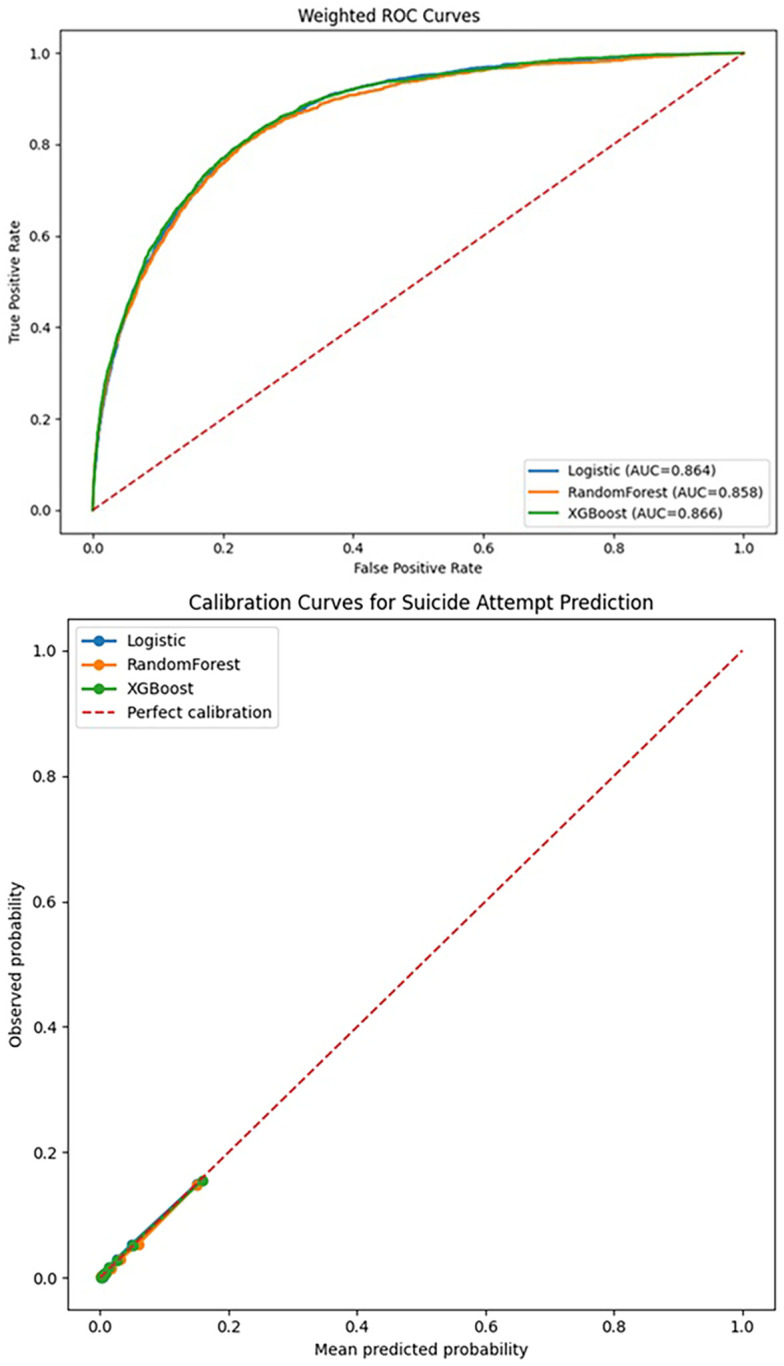
Predictive performance of machine learning models for suicide attempt. **(A)** Receiver operating characteristic (ROC) curves comparing logistic regression, random forest, and XGBoost models. **(B)** Calibration curves showing agreement between predicted and observed probabilities across models.

**Table 3** presents the predictive performance of machine learning models for suicide attempt, including calibration performance measured using the Brier score. **Figure 1** shows the ROC curves and the calibration curves.

**Table 3:** Predictive performance of machine learning models for suicide attempt.

A. F1-score maximization									
Model	Threshold	AUC	Accuracy	Sensitivity	Specificity	PPV	NPV	F1-score	Brier score
Logistic Regression	0.13	0.864	0.953	0.322	0.970	0.230	0.970	0.269	0.0234
Random Forest	0.14	0.858	0.953	0.336	0.970	0.236	0.970	0.278	0.0232
XGBoost	0.16	0.867	0.960	0.300	0.978	0.274	0.978	0.286	0.0231

B. Screening (Recall ≥ 0.70)									
Model	Threshold	AUC	Accuracy	Sensitivity	Specificity	PPV	NPV	F1-score	Brier score
Logistic Regression	0.04	0.864	0.838	0.702	0.842	0.109	0.990	0.188	0.0234
Random Forest	0.04	0.858	0.809	0.745	0.810	0.098	0.990	0.172	0.0232
XGBoost	0.04	0.867	0.837	0.710	0.841	0.109	0.990	0.189	0.0231

Under the F1-score maximization condition, XGBoost showed the highest F1-score (0.29), followed by Random Forest (0.28) and logistic regression (0.27). The area under the curve (AUC) was highest for XGBoost (0.867), followed by logistic regression (0.864) and Random Forest (0.858). Accuracy ranged from 0.95 to 0.96 across all models, while sensitivity ranged from 0.30 to 0.34 and specificity from 0.96 to 0.97. Positive predictive value (PPV) ranged from 0.23 to 0.27, and negative predictive value (NPV) was consistently high at 0.96 across models. Under the sensitivity-oriented threshold (recall ≥ 0.70), sensitivity improved across models, although positive predictive values remained low, indicating a relatively high false-positive rate.

Under the sensitivity-oriented screening condition (recall ≥ 0.70), all models showed increased sensitivity. Random Forest showed the highest sensitivity (0.75), followed by XGBoost (0.71) and logistic regression (0.70). The AUC values remained unchanged. Accuracy ranged from 0.81 to 0.84, and specificity ranged from 0.82 to 0.85. PPV ranged from 0.10 to 0.11, whereas NPV remained high at 0.99 across all models. F1-scores ranged from 0.17 to 0.19. Although sensitivity improved under the screening condition, positive predictive values remained low, indicating a relatively high false-positive rate.

### SHAP-based feature importance

**Figure 2:**
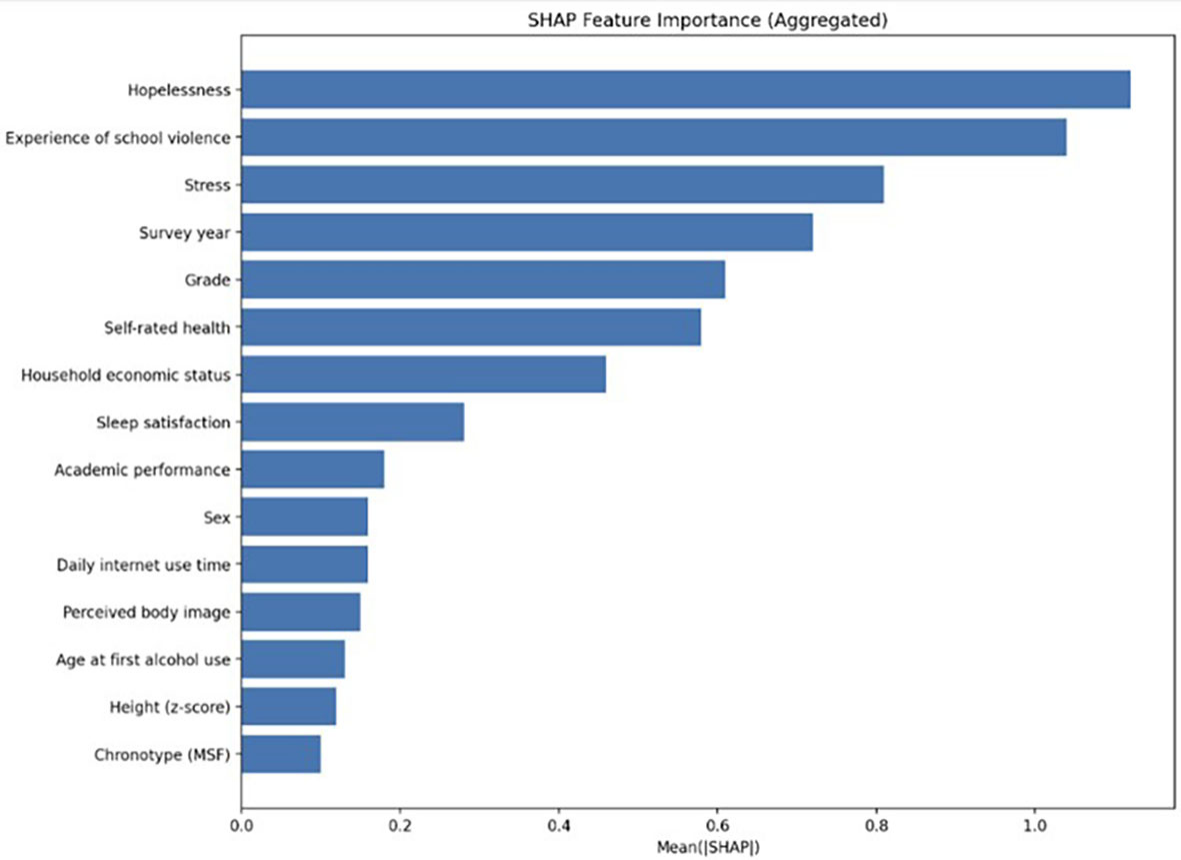
Mean absolute SHAP values indicate the relative importance of each variable in the suicide attempt prediction model.

**Figure 2** presents the SHAP-based feature importance for suicide attempt. Hopelessness showed the highest mean absolute SHAP value, followed by the frequency of school violence experiences and stress. Self-rated health, household economic status, and sleep satisfaction also showed relatively high SHAP values.

## Discussion

This study examined suicide attempts among adolescents using a prediction-oriented framework based on nationally representative data and further explored the relative contribution of predictors using explainable machine learning. Overall, the predictive models demonstrated moderate-to-high discrimination performance, with AUC values exceeding 0.85. In the SHAP-based analysis, hopelessness showed the highest contribution to the prediction of suicide attempts, followed by school violence experiences and stress. Self-rated health, household economic status, and sleep satisfaction also demonstrated relatively high importance. Taken together, the findings provide evidence regarding both the predictive performance of population-based suicide risk models and the relative contribution of individual predictors, thereby advancing understanding of adolescent suicide risk prediction at the population level.

Under the screening condition prioritizing sensitivity, random forest achieved the highest sensitivity (0.75), followed by XGBoost (0.71) and logistic regression (0.70), while positive predictive values remained low. Calibration performance was similar across models. XGBoost showed the lowest Brier score (0.0231), followed by Random Forest (0.0232) and logistic regression (0.0234), indicating slightly better probabilistic calibration performance for XGBoost. The predictive performance observed in the present study was broadly comparable to recent meta-analytic findings reporting pooled AUC values for adolescent suicide prediction models (27). However, despite acceptable discrimination performance, predictive precision remained limited, particularly under sensitivity-oriented screening conditions. First, this study used nationally representative survey data rather than clinically enriched samples, relying primarily on self-reported indicators rather than proximal clinical variables, which are known to substantially influence suicide risk prediction (2). Second, the relatively low base rate of suicide attempts in population-based survey data may inherently limit positive predictive value despite good discrimination performance. This issue is well recognized in rare-outcome prediction models and may contribute to the high false-positive rates observed under sensitivity-oriented screening conditions (2, 28). Third, many previous studies included in meta-analyses relied on internal validation, which may overestimate model performance and limit generalizability (27). Although machine learning approaches can capture non-linear relationships and interactions among predictors, their performance gains over conventional statistical models may be incremental rather than substantial (19). The comparable performance of logistic regression and machine learning models observed in the present study suggests that the relationships between measured risk factors and suicide attempts may be largely captured by conventional statistical approaches. This finding suggests that the predictive information contained in the current set of weighted population-based survey variables may already be largely captured by conventional statistical models, thereby limiting the incremental benefit of more complex machine learning approaches.

Under the sensitivity-oriented threshold (recall ≥ 0.70), model sensitivity improved to approximately 0.70–0.75, but this was accompanied by a low positive predictive value, reflecting a high rate of false positives. Additional threshold analyses demonstrated substantial trade-offs between recall and precision across screening conditions. Lower thresholds improved sensitivity but increased false-positive classifications, whereas higher thresholds improved precision at the expense of recall. Calibration performance was generally comparable across models, with XGBoost showing slightly lower Brier scores, although these differences were modest. This trade-off indicates that the models are more appropriate for population-level screening, where minimizing false negatives is prioritized, rather than for individual-level clinical decision-making (22). From a public health perspective, these models may be most useful as population-level screening tools in school or community settings rather than as diagnostic instruments. Adolescents identified as high-risk through screening could undergo further assessment by school counselors, mental health professionals, or community mental health services. In this context, the primary value of machine learning is not to replace clinical judgment but to facilitate the identification and prioritization of adolescents who may benefit from further evaluation and preventive intervention. Taken together, these findings suggest that machine learning models based on population-level data may serve as useful tools for large-scale risk screening, but they should be interpreted cautiously and used in conjunction with comprehensive clinical evaluation rather than as standalone decision-making tools.

The SHAP-based analysis provided insight into the relative contribution of individual predictors within the model. The present study primarily focused on global feature importance to identify overall population-level predictor contributions. Hopelessness showed the highest contribution, followed by school violence and perceived stress, indicating that proximal psychological and social experiences are strongly reflected in prediction. These findings are consistent with previous research identifying hopelessness and stress as key indicators of emotional vulnerability (4, 5, 11, 12), although the present study interprets these variables in terms of predictive contribution rather than causal effects. The substantial contribution of school violence highlights the importance of repeated interpersonal adversity during adolescence, consistent with prior evidence linking bullying and peer victimization to suicidal behaviors (4, 6). In addition, self-rated health and household economic status contributed meaningfully to the model, suggesting that subjective health status and socioeconomic context may function as integrative markers of vulnerability (5, 10). Sleep satisfaction also contributed to prediction, supporting the potential relevance of sleep-related factors, although its contribution was smaller relative to psychological variables (15, 16). In contrast, some variables such as survey year, height, and internet use time showed statistically significant associations but relatively limited practical contribution, likely reflecting the influence of the large sample size. Overall, these findings suggest that suicide attempt prediction is driven by the interaction of multiple domains, with psychological factors playing a central role, while social and health-related behavioral factors contribute additional predictive value.

This study provides important implications for both research and practice. The findings suggest that machine learning models using large-scale survey data may support population-level screening approaches of adolescents at risk of suicide attempts. Key predictors, including hopelessness, school violence, and stress, highlight the importance of early detection of psychological distress and adverse social experiences, while health-related factors such as self-rated health and sleep satisfaction offer practical signals for risk monitoring.

### Strengths and limitations

This study has several strengths. First, it used large-scale, nationally representative data, enhancing the generalizability of the findings to Korean adolescents. Second, it integrated conventional statistical analysis with machine learning approaches, allowing both the identification of associated factors and the evaluation of predictive performance within a single framework. Third, the application of SHapley Additive exPlanations (SHAP) enabled the interpretation of machine learning models, providing insight into the relative contribution of multiple domains of risk.

However, this study has several limitations. First, the cross-sectional design precludes causal inference, and the identified predictors should be interpreted in terms of predictive contribution rather than causality. Second, all variables were based on self-reported data, which may introduce recall and reporting bias, and social desirability bias. Third, important proximal predictors, such as clinical diagnoses and prior suicidal behaviors, were not available, which may have limited predictive performance. Finally, although the models demonstrated moderate discrimination performance, the relatively low positive predictive values observed under the screening condition indicate a high false-positive rate. Therefore, the models should be interpreted cautiously and may be more appropriate for supportive population-level screening rather than standalone clinical decision-making.

## Conclusion

In conclusion, this study demonstrated that machine learning models using nationally representative survey data can identify adolescents at risk of suicide attempts with moderate predictive performance. Psychological factors emerged as the strongest contributors to prediction, while social and health-related factors provided additional predictive value, highlighting the multidimensional nature of adolescent suicide risk. By integrating predictive modeling with explainable machine learning, this study not only evaluated the utility of population-based suicide risk prediction models but also identified the relative contribution of key predictors. These findings may inform population-level screening and prevention strategies, and future research should continue to refine suicide risk prediction models using diverse data sources and independent validation populations.

## Data Availability

The original contributions presented in the study are included in the article/supplementary material. Further inquiries can be directed to the corresponding authors.
